# Identification of Naturally Occurring Cartilage Damage in the Equine Distal Interphalangeal Joint Using Low-Field Magnetic Resonance Imaging and Magnetic Resonance Arthrography

**DOI:** 10.3389/fvets.2019.00508

**Published:** 2020-01-28

**Authors:** Claudia van Zadelhoff, Tobias Schwarz, Sionagh Smith, Antoine Engerand, Sarah Taylor

**Affiliations:** ^1^Royal (Dick) School of Veterinary Studies and Roslin Institute, The University of Edinburgh, Roslin, United Kingdom; ^2^INSEAD Business School, Fontainebleau, France

**Keywords:** articular cartilage, saline, gadolinium, coffin joint, MRI

## Abstract

**Objectives:** To describe detectable and non-detectable naturally occurring cartilage damage of the equine distal interphalangeal (DIP) joint using plain magnetic resonance (MR) imaging and gadolinium and saline MR arthrography. The second objective was to quantify the sensitivity, specificity and accuracy in detection of cartilage damage.

**Methods:** In a pilot study, the distal limbs of two horses with confirmed osteoarthritis of the DIP joint were imaged with low-field MR. Magnetic resonance images were assessed in consensus by three observers and compared to gross pathological findings. Subsequently, a prospective analytical cross-sectional study design was created to compare pre-contrast MR imaging and saline and gadolinium MR arthrography of isolated equine distal limbs to gross observation findings. Hallmarq® low-field MR (0.27T) scans were performed prior to DIP joint injection, saline/gadolinium post-injection scans were performed at 15 min intervals for 2 h. Joints were inspected and the articular cartilage graded subjectively for cartilage damage (0–3). The presence of detectable or non-detectable cartilage damage on MR images was identified, characterized and recorded in consensus by three observers. Sensitivity, specificity and accuracy in detection of cartilage damage related to gross pathology were calculated.

**Results:** The two clinical cases from the pilot study with confirmed osteoarthritis had full thickness cartilage defects; however, only one of these was correctly identified using low-field MRI. In the prospective study, the majority of naturally occurring cartilage damage could not be identified on plain MR or MR arthrography including extensive partial thickness cartilage erosions. Saline and gadolinium MR arthrography did not improve the detection of cartilage damage. The accuracy of cartilage damage detection was 0.63 with a sensitivity of 0.14 and specificity of 0.92.

**Clinical Relevance:** Both, plain low-field MRI and MR arthrography are not sensitive in detection of naturally occurring cartilage damage of the DIP joint. However, if an abnormal contour is seen in the articular cartilage, cartilage damage is likely to be present.

## Introduction

The distal interphalangeal (DIP) joint is an important source of lameness in sports horses (1) and osteoarthritis plays a major role as a cause for lameness located to this joint (1–3). Articular cartilage injuries are part of the pathophysiological process of osteoarthritis and changes are difficult to detect radiographically (4). The poor healing capacity of articular cartilage makes an early diagnosis critical before irreversible damage occurs (5).

Computed tomography (CT) and magnetic resonance (MR) imaging, has been described to help identify cartilage injury (6–10). Magnetic resonance imaging is widely available in equine practice. Standing low-field MR systems provide 3D imaging to equine patients without the need for general anesthesia. The use of intra-articular contrast injection for MR arthrography may help to increase the visualization of the articular cartilage surface (10, 11). Two major contrast agents are described for MR arthrography: saline and gadolinium (11–13). Saline provides contrast enhancement of synovial structures in fluid sensitive sequences and gadolinium is a paramagnetic metal causing shortening of T1 and T2 relaxation times (14). T1- and T2-weighted sequences have been used for cartilage evaluation in MR imaging and MR arthrography (14, 15). A good contrast has been described between cartilage and the subchondral bone with T1-weighted gradient echo (GRE) sequences. T2-weighted fast spine echo (FSE) sequences are known to provide an accurate high contrast between cartilage (low signal) and joint fluid (high signal) (15, 16). In quantitative cartilage assessment, gadolinium has been injected intra-articularly and cartilage relaxation times measured. A time delay has been shown to be an important factor for measurements in delayed gadolinium enhanced MR imaging (dGEMRIC) (14, 17, 18). A quantitative cartilage assessment has been described for T1- and T2-weighted sequences (T2 mapping) in equine metacarpo- and metatarsophalangeal joints (18). DGEMRIC was recently analyzed in the equine DIP joint using a high-field system and showed relaxation time changes in horses with naturally occurring osteoarthritis (19). The characteristics of naturally occurring cartilage damage that can be identified subjectively in low-field MR and MR arthrography in the equine DIP joint have not been previously described. The aims of this study were to describe detectable and non-detectable naturally occurring cartilage damage of the equine DIP joint using plain low-field MR imaging and gadolinium and saline MR arthrography and to quantify the sensitivity, specificity and accuracy in subjective detection of this damage.

It was hypothesized that full thickness naturally occurring cartilage damage would be identified in low-field MR and that MR arthrography would improve the detection of cartilage damage.

## Materials and Methods

### Pilot Study

Two horses that were subjected to euthanasia at the Royal (Dick) School of Veterinary Studies (R(D)SVS) Equine Hospital, University of Edinburgh during 2017 were included. The first horse presented for clinical lameness of the right fore and left hind limb that was localized to the DIP joint of the right fore foot and the proximal suspensory area of the left hind limb with diagnostic analgesia. Ante-mortem 0.27T low-field open MR system^1^ MR scans of these areas demonstrated signal changes consistent with osteoarthritis of the DIP joint and proximal suspensory ligament desmopathy and enthesopathy of the left hind limb. The second case was subjected to euthanasia for reasons unrelated to lameness however, the horse had a clinical history of recurrent forelimb lameness. The distal limbs were collected after death and imaged using the standard clinical protocol for foot MR scans. The joints were opened and grossly inspected. The MR scans were retrospectively reviewed in a consensus evaluation with three observers.

### Study Design and Case Selection

A prospective analytical cross-sectional study design was used to compare results of pre-contrast MR imaging, and saline and gadolinium MR arthrography at different time points to gross observation findings. Clinical cases that were subjected to euthanasia between December 2017 and June 2018 at the R(D)SVS, Equine Hospital, University of Edinburgh and which had written owners' consent for research purposes were included into the study. Horses were subjected to euthanasia for reasons unrelated to the study and had no standardized lameness examination prior to death. Ethical approval was provided by the veterinary ethical research committee (VERC) of the University of Edinburgh (VERC approval 4.18).

### Image Acquisition

Each limb was scanned at a temperature of 21°C within 12 h of euthanasia. The distal limbs were not frozen before imaging and were stored at 4°C after scanning until gross observation.

A standard protocol for a 0.27T low-field open MR system^1^ was used for all limbs included in the study. The protocol consisted of T1-weighted GRE high resolution (HR) and T2-weighted FSE HR scans each in dorsal and sagittal planes (Table 1). The pixel size was 0.35 mm in row and column in a field of view of 180 mm. For MR arthrography, the limbs were removed from the magnet and the dorsal recess of the DIP joint was injected with 10ml of saline (0.9% NaCl, Vetivex1)^2^ or gadolinium mixture. The limbs were all flexed 30 times to ensure dissipation of contrast media before repeat MR imaging. The contrast agent was randomly assigned to the limbs using a randomization function (Excel). The gadolinium mixture contained 4.69 mg of gadolinium (gadopentetate dimeglumine, Magnevist®)^3^ (17) and 60 mg of iodine (iomeprol, Iomeron®)^4^ per ml. Iodine was added to the gadolinium mixture to scan the limbs computed topographically for a further project. The limbs were scanned prior to contrast injection and post-contrast scans were acquired in 15 min intervals for 2 h.

**Table 1:** High resolution (HR) pulse sequences parameters using low-field open (0.27T) MRI system^1^.

**Pulse sequence**	**Orientation**	**TE** **(ms)**	**TR** **(ms)**	**FOV** **(mm)**	**Slide width** ** (mm)**	**Gap** ** (mm)**	**Scan time**	**pixel size** ** (mm)**
T1-w GRE	Dors/sag	8	104	180	3.5	0.4	2 min 41 s	0.35
T2-w FSE	Dors/sag	87	1980	180	3.5	0.4	5 min 25 s	0.35

*GRE, gradient echo; FSE, fast spin echo; Dors, dorsal; sag, sagittal; MRI, magnetic resonance imaging; TE, echo time; TR, repetition time*.

### Image Evaluation

The MR images of the pilot study were evaluated in consensus by a Diplomate of the European College of Veterinary Surgeons (Dipl ECVS), a Diplomate of the European College of Veterinary Diagnostic Imaging (Dipl ECVDI) and a veterinary surgeon training in diagnostic imaging. MR images of 32 limbs of 12 horses were assessed in T1-weighted and T2-weighted sequences in dorsal and sagittal planes in 6 locations per limb at one time point prior and two time points post contrast injection (immediately after injection and 120 min post injection) resulting in 1,152 instances. Evaluation sessions with the observers were performed for the prospective study, where 50 scans were graded separately and independently from each other. Images were presented blinded and in a random order, limbs were assigned codes for unbiased scoring. T1- and T2-weighted sequences were evaluated separately, whereas dorsal, and sagittal planes of each sequence were evaluated in combination. All observers evaluated scans separately prior to contrast injection and immediately after contrast injection. A consensus evaluation among the three observers was reached regarding the impact of time delay on gadolinium-injected T1-weighted GRE scans and saline-injected T2-weighted FSE scans. Due to the thin cartilage and the slice orientation in the present study, the articular aspect of the navicular bone was not included in MR evaluation. In dorsal scans, the image orientation was parallel to the deep digital flexor tendon at the level proximal to the navicular bone which makes the articular surface of the navicular bone difficult to assess. The articular cartilage of the distal phalanx (P3) and the middle phalanx (P2) was divided in three equal parts each for adequate damage localization (medial, axial, lateral). For each of these locations an “abnormal contour” “yes” or “no” was assigned. No training was provided beforehand. Data were recorded in a spread sheet (Excel)^5^.

### Macroscopic Cartilage Assessment

After image acquisition, each DIP joint was opened within 24 h of euthanasia by a circumferential cut above the coronary band. The articular surface of P3 and P2 was inspected for macroscopically visible cartilage damage. The term cartilage damage includes both, defects and cartilage degeneration without loss of substance. Cartilage defects were defined as loss of cartilage substance, furthermore cartilage color and smoothness were recorded for each joint and compared to MR images. Each of the six locations was assigned a grade between zero and three; grade 0 corresponded to an intact cartilage surface, grade 1 to focal cartilage defects (diameter <5 mm), grade 2 to large or diffuse defects, fissures, erosions or more than 2 focal defects in the same location of the cartilage (diameter >5 mm) and grade 3 to full-thickness cartilage defects with involvement of the subchondral bone (20, 21). The first author evaluated the specimen after opening and digital pictures were taken for record. Using these photographs, a consensus grading was finally performed by a Diplomate of the American College of Veterinary Pathologists (Dipl ACVP) and the first author.

### Microscopic Cartilage Assessment

The articular cartilage of P3 of three limbs of three different horses was analyzed. These individuals with grade 2 defects were subjectively chosen for confirmation where macroscopic cartilage damage was previously identified. The specimens were fixed in 10% neutral buffered formalin for 48–72 h, decalcified in Decal I (methanol, formaldehyde and formic acid)^6^ until sufficiently soft to cut (~7–10 days depending on sample) and embedded in paraffin wax. Four-micron thick sections were cut and routinely stained with hematoxylin and eosin (assessed by S.S., Dipl ACVP). The histopathological findings were described in detail and subjectively compared to macroscopic findings. No grading system for microscopic cartilage assessment was used as only three specimens were available.

### Statistics

Descriptive statistics were used to describe the number, grade and location of cartilage defects identified by MR imaging and gross observation. The sensitivity, specificity and accuracy in detection of cartilage defects in T1- and T2-weighted sequences was calculated for pre-contrast scans and immediate post-contrast scans after injection of saline or gadolinium with regards to the corresponding defects in gross observation. The accuracy was calculated by division of the sum of true positives (identified in MR and cartilage defect present in gross observation) and true negatives (no defect identified in MR and cartilage without defect in gross observation) over the total number of assessed locations. Analyses were performed using a statistical software (R)^7^.

## Results

### Pilot Study

Horse 1 was a 6-year-old Highland Pony gelding and horse 2 was a 12-year-old Thoroughbred gelding.

**Figure 1:**
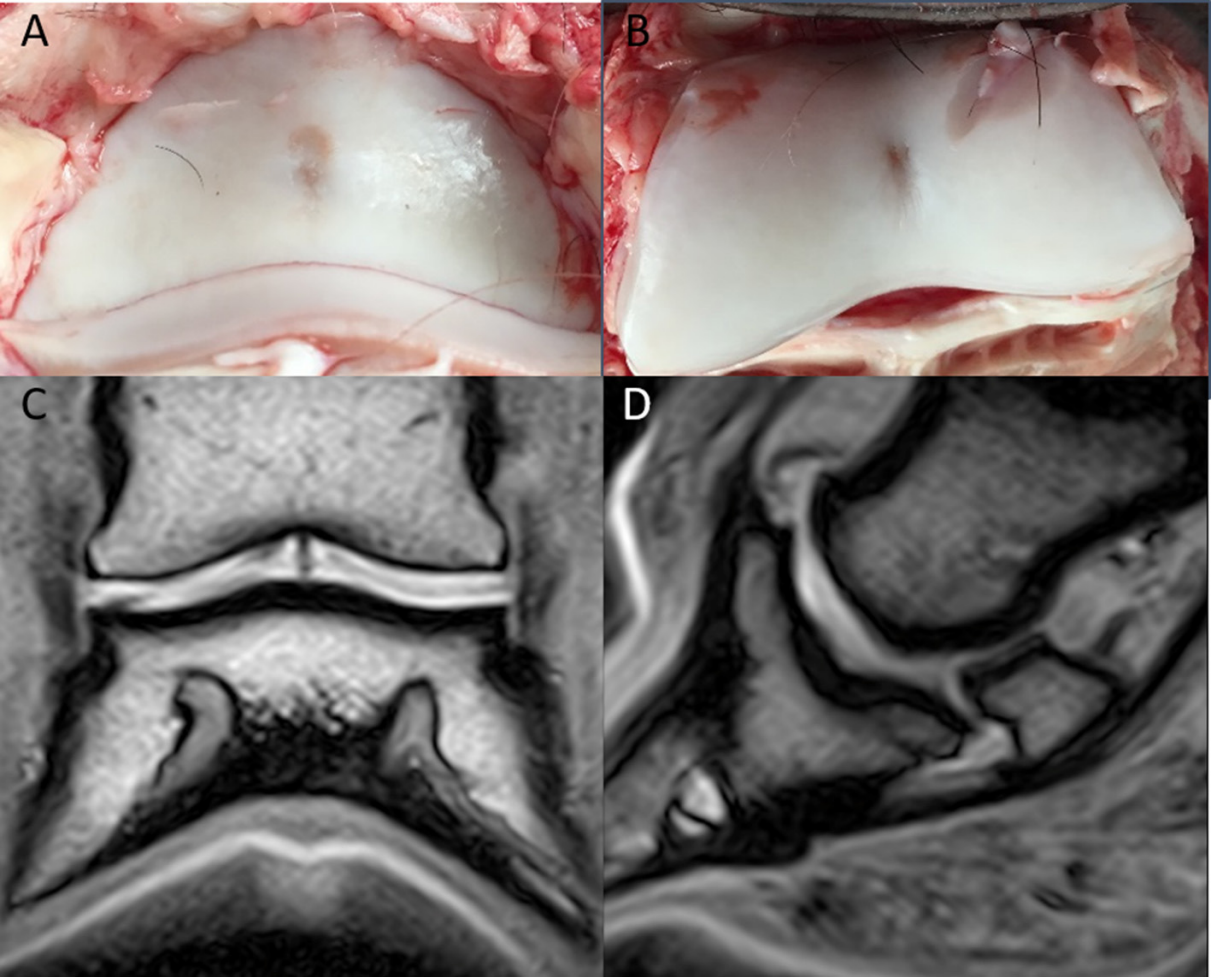
Clinical case from the pilot study, horse one. Medial is to the left. In the axial aspect of the distal phalanx **(A)**, and the middle phalanx **(B)**, there is a focal cartilage defect. Note the sharp-edged artifactual cut into the cartilage during preparation **(B)**; Dorsal **(C)**, and sagittal **(D)** T1-weighted sequences in plain low-field MR imaging. An irregular joint space and a hypointense signal are visible in the axial aspect of the articular cartilage of the distal and middle phalanx.

**Figure 2:**
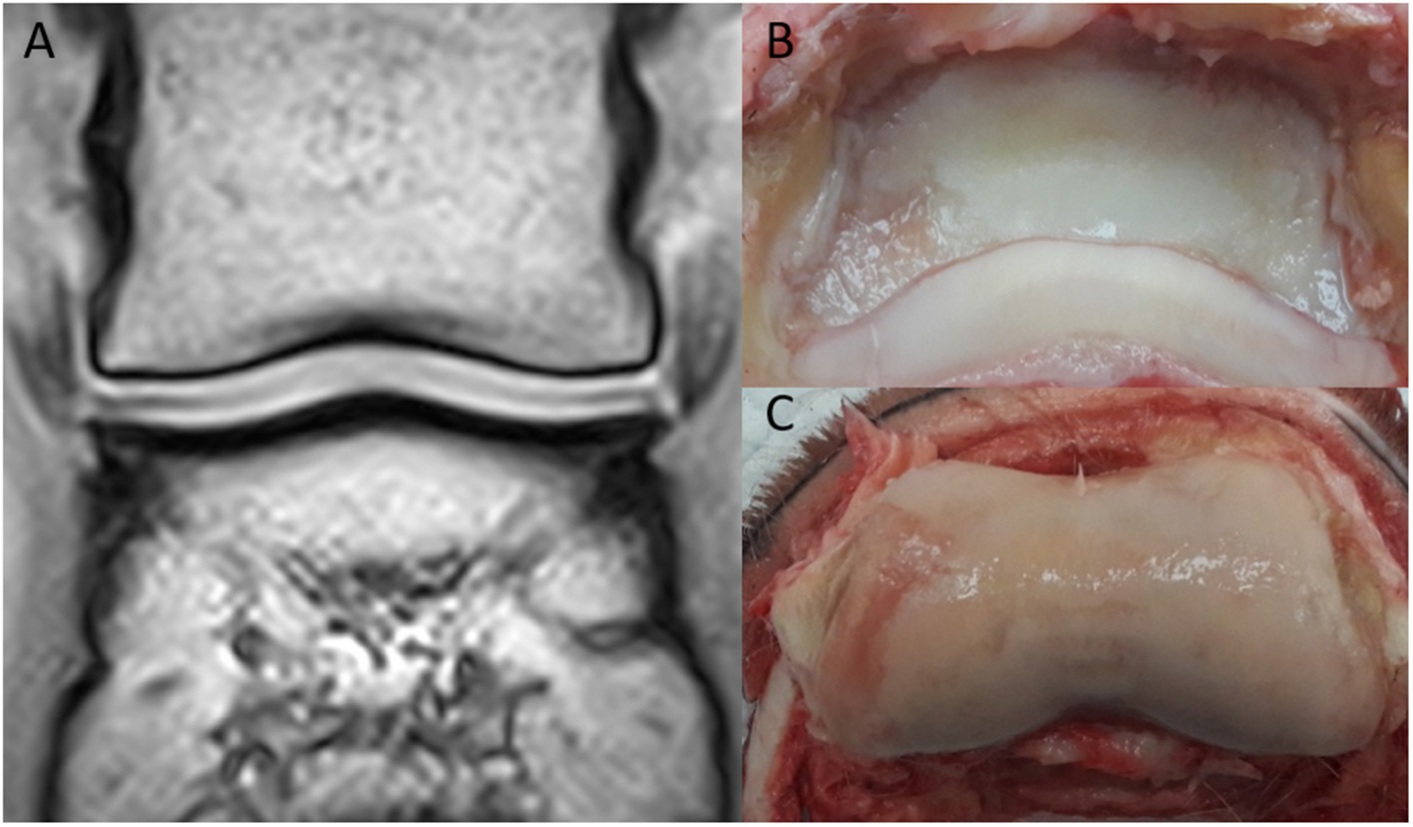
Clinical case from the pilot study, horse two. Medial is to the left. **(A)** Dorsal T1-weighted sequences in plain low-field MR, no significant abnormalities were identified. In the medial and lateral aspect of the distal phalanx **(B)**, there is cartilage erosion (more pronounced medially). There is a full-thickness cartilage defect medially in the middle phalanx **(C)**, with yellow/gray discoloration of the articular cartilage.

The MR images of the two isolated distal limbs of these two clinical cases were assessed in consensus by the three observers and cartilage damage was clearly identified in one of the two cases. Gross post mortem inspection of the cartilage confirmed the damage was in the same location as noted on MR (Figure 1). However, marked cartilage defects were seen on gross pathology in case two, which could not be identified on MR images, even retrospectively (Figure 2).

### Case Selection

Twenty front feet and twelve hind feet were collected from twelve horses. The median age of the horses was 13.5 years old [inter quartile range (IQR) = 9.5 years]. Six geldings and six mares of different breeds [Thoroughbred (*n* = 6), Warmblood/Warmblood cross (*n* = 3), Welsh cob (*n* = 1), ponies (*n* = 2)] were included in the study.

### Macroscopic Cartilage Assessment

A total of 72 cartilage defects were identified in 192 locations in 25/32 limbs (78.13%). A grade 1 was assigned to 11/72 cartilage defects and a grade 2 to 61/72, respectively. A grade 3 was not identified in any of the locations. Most of the defects were located in the P3 lateral location (16/72), followed by location P2 lateral (13/72) and the P2 medial (13/72). Fewer defects were identified in location P3 medial (11/72), P3 axial (9/72) and P2 axial (10/72). Twenty fore limbs and twelve hind limbs were included into the study and half of the fore limbs and hind limbs were injected with gadolinium and the other half with saline. The cartilage defect locations of all limbs are summarized in Table 2.

**Table 2:** Cartilage defects in 32 limbs (20 fore limbs, 12 hind limbs) on macroscopic cartilage evaluation by location.

**Cartilage defect**	**P3med**	**P3ax**	**P3lat**	**P2med**	**P2ax**	**P2lat**
Grade 0	21	23	16	19	22	19
Grade 1	0	4	1	2	3	1
Grade 2	11	5	15	11	7	12

*A grade 3 was not identified in any location*.

*P3, distal phalanx; P2, middle phalanx; med, medial; ax, axial; lat, lateral; grade 0, intact cartilage surface; grade 1, focal cartilage defects (diameter <5 mm), grade 2 = large or diffuse defects, fissures, erosions or more than 2 focal defects in the same location of the cartilage (diameter >5 mm); grade 3, full-thickness cartilage defects with involvement of the subchondral bone*.

### MR Evaluation and Comparison to Gross Observation and Microscopic Cartilage Assessment

Each blinded observer evaluated independently T1-weighted GRE and T2-weighted FSE sequences in dorsal and sagittal planes of the 32 limbs and noted cartilage abnormalities “yes” or “no”. However, the observers could rarely see a defect on MR scans and did not feel confident in correctly identifying cartilage defects. The sensitivity, specificity, and accuracy of the evaluation from observer one were 0.20, 0.89, and 0.63, respectively. Observer two gradings had a sensitivity of 0.17, specificity of 0.91, and accuracy of 0.63. Observer three gradings had a sensitivity of 0.04, specificity of 0.96, and accuracy of 0.62. The true negative, true positive, false negative, and false positive values regarding the detection of cartilage defects grade 1 and grade 2 are provided in Table 3. The overall sensitivity was 0.14, specificity 0.92, and accuracy of 0.63. These values did not improve with MR arthrography or time delayed imaging. A subsequent consensus evaluation was performed, where all three observers decided that the use of contrast either saline or gadolinium did not improve the detection of cartilage defects and that there was no change of MR arthrography images with time delayed imaging (Figure 6). The observers found T2-weighted FSE sequences to be more useful in detection of cartilage abnormalities compared to T1-weighted GRE sequences. In separate evaluation, the T2-weighted sequences were more sensitive and mildly less specific in detection of cartilage defects (sensitivity 0.21, specificity 0.87) compared to T1-weighted sequences (sensitivity 0.06, specificity 0.96).

**Table 3:** Sensitivity, specificity, accuracy, and true positive/true negative/false positive/false negative values per observer regarding cartilage defect size (grade 1, grade 2) in evaluation of a total of 1,152 locations (32 joints × 6 locations × 2 sequences × 3 time points).

**Observer**	**Sensitivity**	**Specificity**	**Accuracy**	**True** **neg**	**False** **neg**	**False** **neg**	**False** **pos**	**True** **pos**	**True** **pos**
					**Grade 1**	**Grade 2**		**Grade 1**	**Grade 2**
1	0.20	0.89	0.63	638	59	287	82	7	79
2	0.17	0.91	0.63	655	51	307	65	15	59
3	0.04	0.96	0.62	693	61	354	27	5	12

*Sensitivity: sum of true positives divided by sum of true positives and false negatives; specificity: sum of true negatives divided by sum of true negatives and false positives; accuracy: sum of true positives and true negatives divided by 1,152*.

*Neg, negative; pos, positive; grade 1, cartilage defects grade 1 (<5 mm), grade 2, cartilage defects grade 2 (>5 mm)*.

**Figure 3:**
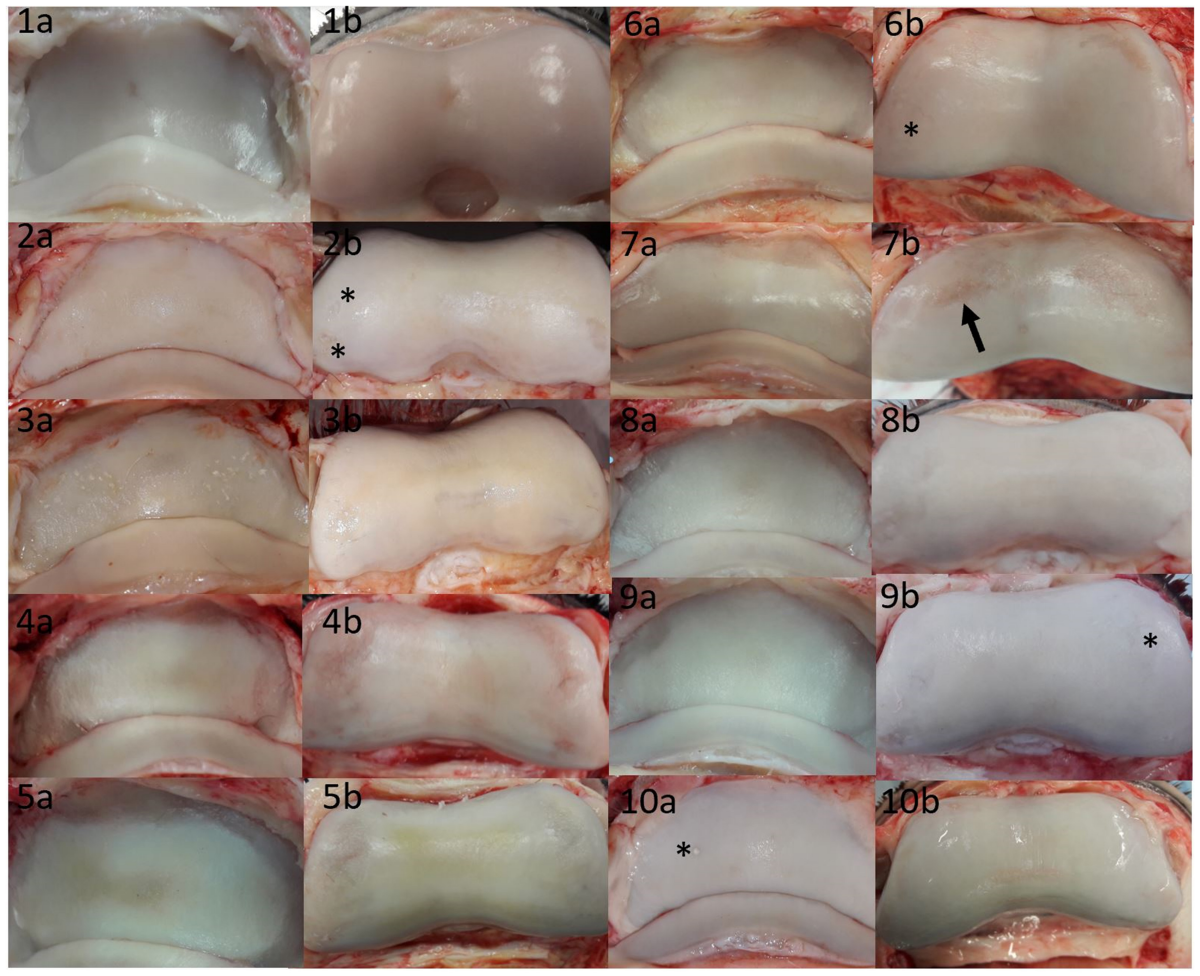
Summary of cartilage damage that could not be identified in low-field magnetic resonance or magnetic resonance arthrography. Lateral is to the left; images labeled **(a)** are P3, and images labeled **(b)** are P2 pictures of the same joint. There was mild yellow/gray discoloration and roughening of the articular cartilage of all bones, with the exception of **(1a,1b,9b,10a)**. **(1a)** Focal grade 1 cartilage defect in axial P3; **(1b)** focal grade 1 cartilage defect in axial P2; **(2a)** mild diffuse yellow discoloration and roughening over >50% of the articular cartilage; **(2b)** grade 2 cartilage defects in medial and lateral (asterisks) P2; **(3a)** grade 2 defects in P3 medially and laterally [note that in the contralateral limb with similar changes, signal changes could be seen on MR (Figure 4)]; **(3b)** grade 2 defects on the axial and medial surfaces of P2; **(4a)** grade 2 defect in P3 medially and laterally; **(4b)** grade 2 defect in P2 medially and laterally; **(5a)** grade 2 circumferential sudden cartilage thinning in P3 medially, axially and laterally; **(5b)** grade 2 defect in P2 medially and laterally; **(6a)** sudden cartilage thinning medially and laterally in P3, scored as grade 2; **(6b)** grade 2 defect in P2 medially and laterally (asterisk); **(7a)** grade 2 defect in P3 spanning the transition between axial and medial; **(7b)** grade 2 defect in P2 medially and laterally, note that the lateral defect (arrow) could be identified in MR (Figure 5); **(8a)** subtle grade 1 defect in P3 axially and grade 2 defects in P3 medially and laterally; **(8b)** grade 2 defects P2 medially and laterally; **(9a)** grade 2 defect in P3 laterally; **(9b)** grade 2 defect in P3 laterally and medially (asterisk); **(10a)** grade 1 mineralization defect in P3 lateral (asterisk), **(10b)** grade 2 defect in axial P2 (wear lines visible) surrounded by focal roughness and off-white discoloration>50%.

**Figure 4:**
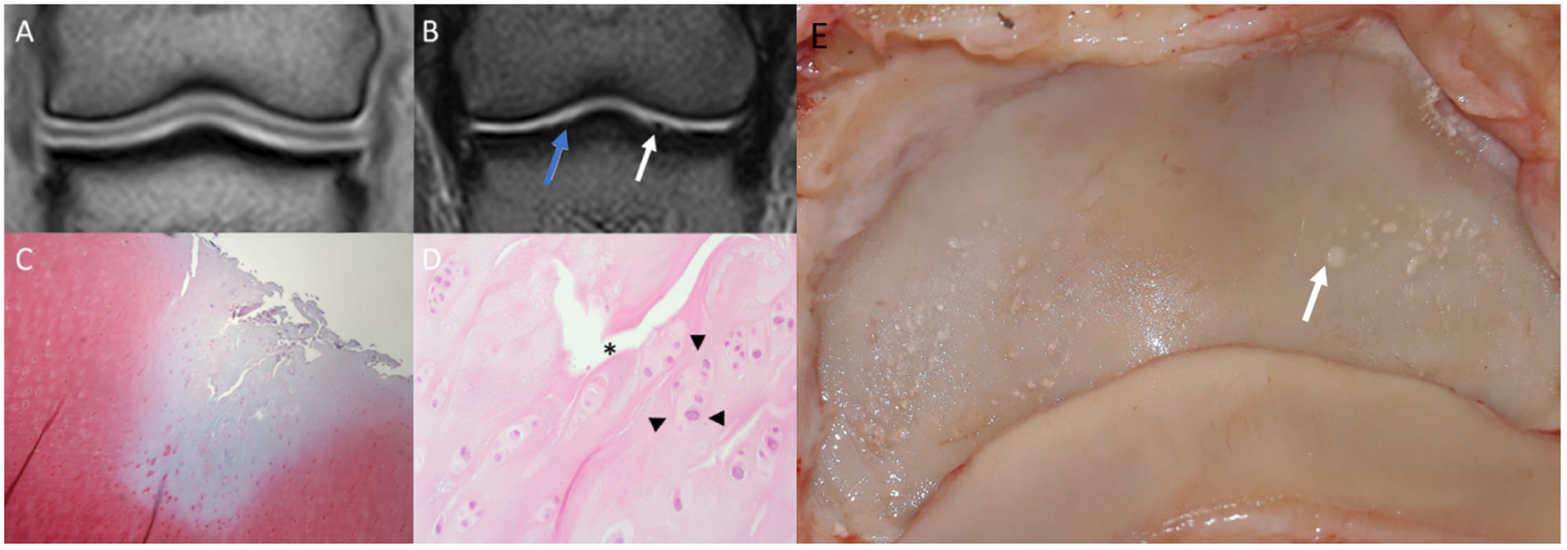
Saline magnetic resonance (MR) images, histology and gross inspection images of the same horse. Lateral is to the left. **(A)** Dorsal T1-weighted image. Note negative contrast post saline injection as hypointense signal compared to the adjacent hyperintense cartilage signal. **(B)** Dorsal T2-weighted FSE image. Note the irregular outline (white arrow) of the medial aspect of the articular surface of the distal phalanx (P3). There is a mildly irregular outline of the articular cartilage laterally (blue arrow), which may represent cartilage damage or volume averaging artifact on a curved surface. **(C)** Safranin-O fast green staining of a macroscopically fibrillated area; the blue discoloration indicates a reduction in proteoglycans. **(D)** Perpendicular fissures in the superficial cartilage (asterisk) and an abnormal clumping (“chondrones”) of chondrocytes (arrowheads), HE. **(E)** Gross inspection of the distal phalanx. There are multiple focal cartilage defects medially and laterally. The white arrow corresponds to the white arrow in **(B)**.

**Figure 5:**
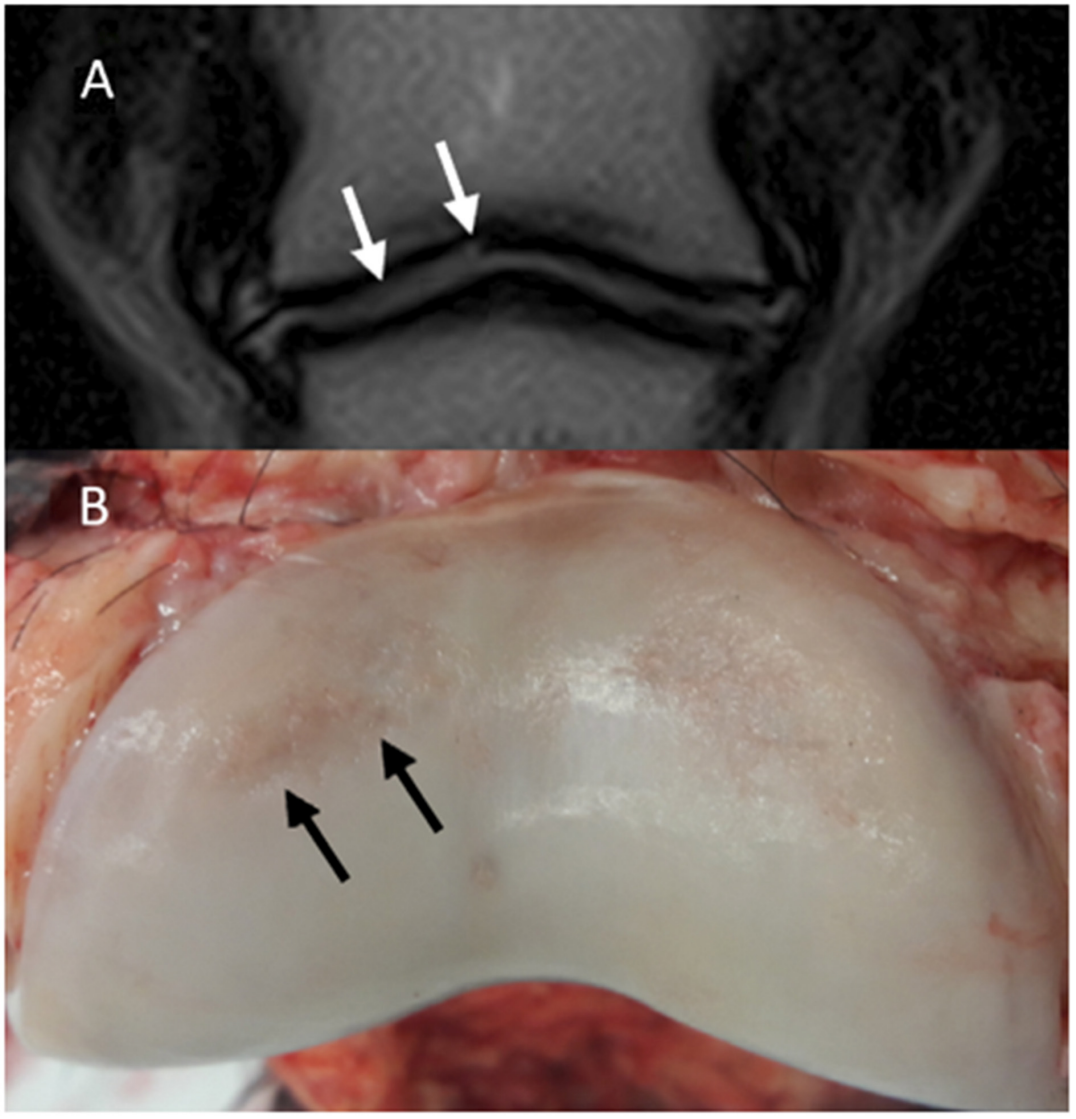
Magnetic resonance (MR) and macroscopic images of the same horse. Lateral is to the left; **(A)** Dorsal T2-weighted FSE. A hyperintensity is noted in the lateral aspect of the articular cartilage of the middle phalanx (P2) (white arrows). **(B)** Gross appearance of P2. There are cartilage erosions in the dorsolateral (black arrows) and dorsomedial aspects of the articular surface. The cartilage defect on the dorsomedial aspect appears more superficial compared to the dorsolateral aspect and could not be identified in MR images.

As few defects could be detected on MR scans, the authors assessed the macroscopic cartilage defects retrospectively, to collect a variety of damage which were missed in MR image evaluation (Figure 3). This damage included several large partial thickness defects, focal and generalized yellow discoloration, sudden thinning within the cartilage, focal mineralization, a generalized roughened surface, and cartilage fibrillation. Examples of correctly identified cartilage defects are shown in Figures 4, 5. The MR findings were compared to the location of the macroscopic cartilage defect and in three cases of grade 2 cartilage defects a histopathologic examination was available. In all three cases histopathologic results confirmed macroscopic findings. A summary of the 32 analyzed limbs with the assigned contrast group and macroscopic cartilage grades per location is provided in Supplementary Table 1.

**Figure 6:**
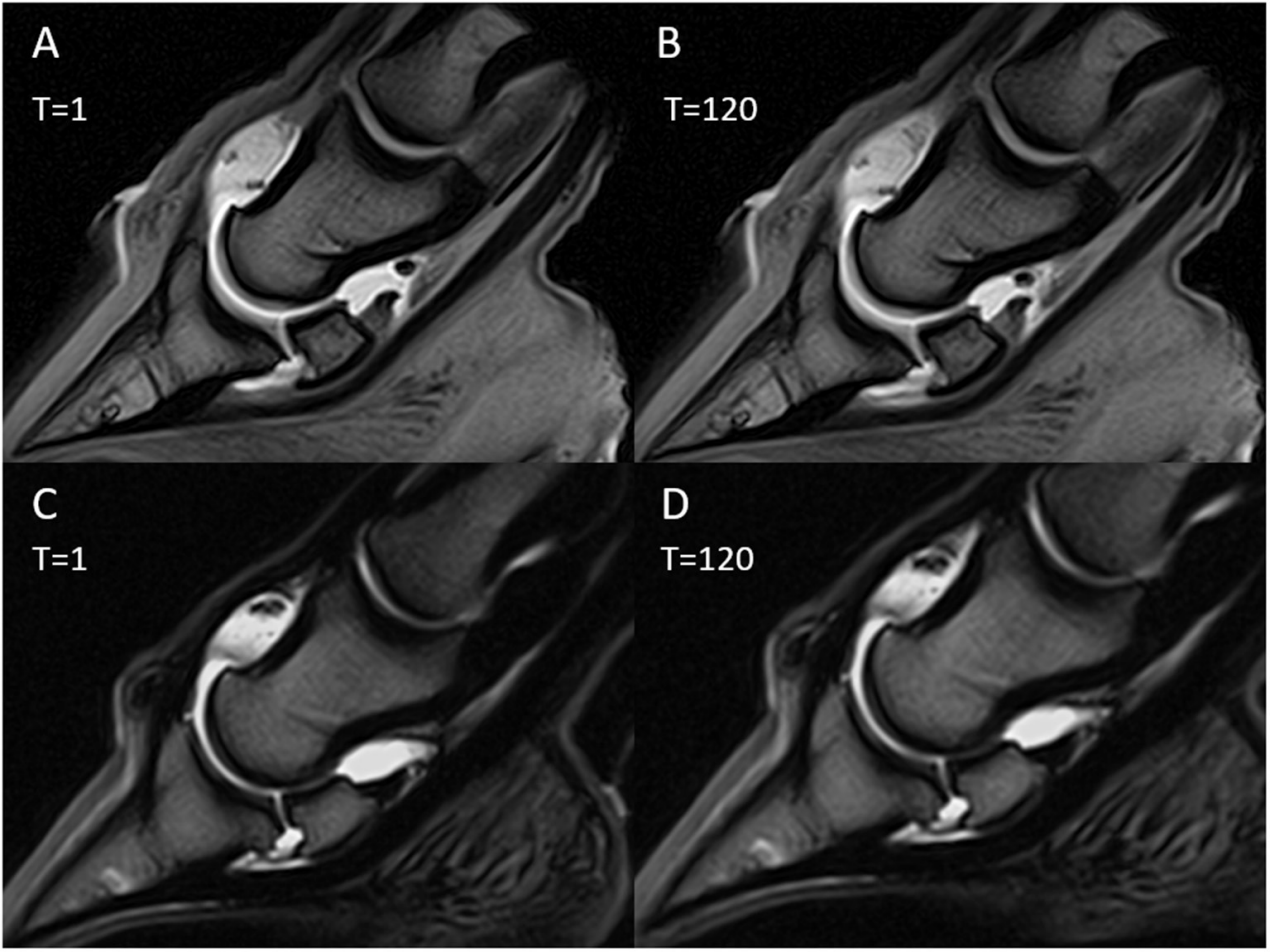
Magnetic resonance (MR) arthrography images immediately after contrast medium injection (*T* = 1) and after 120 min (*T* = 120). Sagittal T1-weighted images with gadolinium injection **(A,B)**; sagittal T2-weighted images with saline injection **(C,D)**.

## Discussion

Our prospective study has shown that low-field MRI without and with arthrography is rarely able to detect naturally occurring cartilage damage in the DIP joint in horses. Detection of cartilage damage with MR imaging has been described in high-field MRI in the equine carpal joint (22), the metacarpophalangeal joint (8) and the DIP joint (19) and in low-field MRI of the DIP joint (16). The sensitivity in detection of cartilage damage was lower in this study compared to previous studies in low-field MRI (16, 23), where cartilage defects have been created manually have been created manually. These defects with sharp margins might be identified more easily compared to naturally occurring cartilage damage. The detection of cartilage damage has been reported to be better in high-field MRI when compared to low-field MRI (15, 23, 24). Lower resolution due to increased slice thickness leading to increased partial volume averaging in low-field MRI result in a reduced detection of cartilage damage. In our sample population with only superficial cartilage damage, the detection of cartilage damage was limited.

T2-weighted FSE sequences were found to be more sensitive compared to T1-weighted GRE sequences in low-field MRI in detection of cartilage damage of the DIP joint. A previous study described the T2-weighted FSE sequence as most useful in low-field MRI for the detection of cartilage defects (23). One study hypothesized that damaged cartilage contains an increased amount of free water content when compared to normal cartilage which leads to an increased signal intensity in T2-weighted FSE sequences but not in T1-weighted GRE images (23). It has been described that in T1-weighted GRE images the osteochondral surface adjacent to synovial fluid may appear intact due to truncation artifact which then leads to false negative results (15, 23, 24). The contrast between cartilage and synovial fluid in GRE sequences is low (25) and GRE is most sensitive to truncation effects (26). All these effects may lead to false negative MR findings which were seen commonly in the present study when evaluating T1-weighted GRE images. On the other hand, T1-weighted GRE images are the only sequence where the cartilage itself can be visualized (15). In T2-weighted FSE the cartilage signal was low, but chondral defects and synovial fluid are hyperintense which may make this sequence more sensitive compared to T1-weighted GRE sequences (15). However, in general the sensitivity in detection of cartilage damage in low-field MR was low in this study with T1-weighted and T2-weighted images.

In contrast to our hypothesis, the sensitivity in detection of cartilage damage did not increase with the injection of saline or gadolinium into the DIP joint. Gadolinium shortens relaxation time in T1-weightes sequences and has only minimal effect in T2-weighted sequences. In low concentration, gadolinium causes a hyperintense signal in T1-weighted sequences; however, in high concentrations it can create a signal void due to susceptibility artifact (14). Improved sensitivity in high-field MRI in detection of artificially created cartilage defects in the equine carpal joint with the use of gadolinium contrast has been reported (22), however this improvement was not statistically significant. Saline arthrography and saline podotrochlear bursography have been described to be helpful to assess the palmar aspect of the navicular bone, the dorsal border of the deep digital flexor tendon and adhesions between the soft tissues of the podotrochlear apparatus and the fibrocartilage of the navicular bone (12, 27). A positive effect of saline arthrography of the equine DIP joint in low-field MRI to evaluate the articular cartilage surface has not been described before and could not be confirmed in the current study. In general, the use of contrast media in MR for cartilage imaging is controversially discussed. One study identified that the detection of metacarpophalangeal or metatarsophalangeal articular cartilage pathology was not increased with the addition of intra-articular contrast media to MR or CT studies (8). Additionally, Porter et al. could not confirm an improved correlation between MR and CT arthrography and gross measurements of cartilage thickness (28). Regarding the contrast agents, Helms et al. tested the diagnostic accuracy of MR arthrography with gadolinium and with saline in the diagnosis of rotator cuff tears and labral defects in the human shoulder and concluded that saline provided equivalent diagnostic information when compared to gadolinium enhancement (29). In human and porcine cartilage, there was no advantage to the use of gadolinium MR arthrography over saline MR arthrography (13, 30). In the present study, neither of the tested intra-articular contrast media, provided better results than the other.

Osteoarthritis is an inflammatory and degenerative condition of diarthrodial joints, one of the hallmarks of osteoarthritis is loss of articular cartilage. Cartilage damage are a feature of osteoarthritis and other chondral diseases. Damage within the cartilage are likely to progress over time which makes them clinically relevant (31). In the present study, a variety of cartilage damage was observed in macroscopic evaluation of the DIP joints. This included extensive partial thickness defects, focal and generalized yellow discoloration, sudden thinning or step formations (mainly abaxially), focal mineralization within the cartilage, a generalized roughened surface and cartilage fibrillation. The majority could not be identified on MR images. Only the most profound defects located within the center of the joint were identified on MR. Cartilage defects may be seen in MR when they are profound enough and have sharp margins. However, this was rare in the naturally occurring cartilage damage of the current study and even moderate step formations on the abaxial aspects of the DIP joint could not be identified on MR scans. In the present study, there were mainly partial thickness cartilage defects present, even if these represented extensive cartilage erosions. The nature of cartilage damage in our population and the associated low sensitivity in detection of this showed that low-field MR is not suitable to detect this kind of cartilage damage. However, the high specificity suggests that an abnormal contour in the articular cartilage in MR scans, suggestive of a cartilage defect, are likely to be real.

In the pilot study, two clinical cases with confirmed osteoarthritis of the DIP joint had extensive cartilage erosions on gross inspections of the joints. Only one of the two was obvious on MR scans. The detectable lesion was in the center of the joint, was focal and had abrupt margins which may have helped to create marked cartilage contour changes in low-field MR. The extensive erosions in the second horse which were located in the abaxial margins of the DIP joint and smoothly thinned margins could not be detected on MR. These findings let to the question if low-field MRI can indeed consistently identify cartilage damage in the DIP joint of the horse.

The main limitations of this study were that images were acquired from cadaver limbs and the limbs were not loaded. Results might differ in an *in-vivo* study with weight-bearing horses, where the accuracy might be lower. However, there are clinics using low-field MR under general anesthesia where the limbs are in a non-weight bearing position. As performed by others (8, 12, 15, 16, 18–20, 22, 23, 27, 32), this cadaver study allowed gross inspection of cartilage lesions and histopathology. A referral population of horses was analyzed which is not representative for the normal horse population. Horses were presented to the referral center for various reasons. A further limitation is that the consensus evaluation of macroscopic cartilage damage was performed on digital photographs and the damage depth could macroscopically not be identified. Histology was only performed in a limited number of cases due to financial constraints. The statistical results of the present study are difficult to compare to other studies as the analyzed anatomy, the used magnetic field strength and the positioning were different compared to previous studies.

In conclusion, naturally occurring cartilage damage is common in the DIP joint and presented in the present study with a large variety. A majority of this damage including partial thickness defects could not be detected on low-field MR images. The low sensitivity and high specificity suggest that a normal articular cartilage contour does not mean that the cartilage is healthy. Furthermore, if an abnormal articular cartilage contour is observed and suggestive of a cartilage damage, it is likely to be real. Deep and sharply marginated cartilage defects are more likely to be identified in MR scans. Low-field MR arthrography and time delayed imaging did not improve the accuracy in detecting defects.

## Data Availability Statement

The datasets generated for this study are available on request to the corresponding author.

## Ethics Statement

School of Veterinary Medicine Ethical Review Committee (VERC) of the University of Edinburgh. Clinical cases that were subjected to euthanasia between December 2017 and June 2018 at the Equine Hospital of the University of Edinburgh and which had written owners' consent for research purposes were included into the study. Ethical approval was provided by the veterinary ethical research committee (VERC) of the University of Edinburgh (VERC approval 4.18).

## Author Contributions

CVZ, ST, and TS were involved in design of the study. CVZ performed data acquisition and prepared the manuscript. CVZ, TS, ST, and SS were involved in data interpretation. CVZ and AE were involved in statistical data analysis and interpretation. All authors revised the manuscript and gave their final approval.

### Conflict of Interest

The authors declare that the research was conducted in the absence of any commercial or financial relationships that could be construed as a potential conflict of interest.

## Footnotes

^1^Hallmarq Veterinary Imaging Ltd, Surrey, UK.

^2^Dechra Pharmaceuticals Plc, Lostock Gralam, Northwich, Cheshire, UK.

^3^Bayer plc, Berkshire, UK.

^4^Bracco UK Ltd, Buckinghamshire, UK.

^5^Microsoft, Redmond, WA, USA.

^6^Leica Biosystems, Richmond Inc, Richmond, Illinois, USA.

^7^R Development Core Team (2008). R: A language and environment for statistical computing. R Foundation for Statistical Computing, Vienna, Austria. ISBN 3-900051-07-0, URL http://www.R-project.org.

## Supplementary Material

Supplementary Table 1 Summary of the 32 analyzed limbs with the assigned contrast group and macroscopic cartilage grades per location.
