# Supplementary material for: Identification of Naturally Occurring Cartilage Damage in the Equine Distal Interphalangeal Joint Using Low-Field Magnetic Resonance Imaging and Magnetic Resonance Arthrography
Source: Front Vet Sci. 2020 Jan 28;6:508. doi: 10.3389/fvets.2019.00508 (PMC6999043; doi:10.3389/fvets.2019.00508)
Supplement: Supplementary Table 1 — Summary of the 32 analyzed limbs with the assigned contrast group and macroscopic cartilage grades per location. [file Table_1.docx]

Supplementary Table: Summary of the 32 analyzed limbs with the assigned contrast group and macroscopic cartilage grades per location.

No Limb Contrast P3medP3ax P3lat P2medP2ax P2lat

1 F gadolinium 0 0 0 0 0 0

2 F saline 0 0 1 0 2 0

3 F gadolinium 2 0 0 1 1 0

4 F saline 0 0 0 0 2 0

5 F gadolinium 0 0 0 2 2 0

6 H gadolinium 0 1 0 0 1 0

7 F gadolinium 0 0 0 0 0 0

8 H gadolinium 0 0 2 0 0 0

9 F saline 0 1 2 0 0 2

10 F saline 0 0 0 2 0 2

11 F saline 2 0 2 0 2 0

12 F saline 2 0 2 0 2 2

13 H saline 2 0 2 0 0 0

14 H saline 2 2 2 2 0 0

15 F gadolinium 2 2 2 0 0 0

16 F gadolinium 0 0 0 0 0 0

17 H saline 0 0 0 0 0 0

18 H gadolinium 0 0 0 0 0 0

19 H saline 0 0 2 2 2 2

20 H gadolinium 0 0 0 2 2 2

21 F gadolinium 2 0 2 2 0 2

22 F gadolinium 2 2 2 2 0 2

23 H gadolinium 2 0 2 2 0 2

24 H gadolinium 0 2 2 2 0 2

25 F saline 0 0 0 0 0 0

26 F gadolinium 0 0 0 1 0 0

27 H saline 0 2 0 2 1 1

28 H saline 0 1 0 0 0 2

29 F saline 0 0 0 0 0 0

30 F saline 2 0 2 0 0 0

31 F gadolinium 2 1 2 2 0 2

32 F saline 0 0 2 0 0 2

_____________________________________________________________

F = fore limb; H = hind limb; P2 = middle phalanx; P3 = distal phalanx, ax = axial; med = medial; lat = lateral.
